# The Association Between the Number of Consecutive Night Shifts and Insomnia Among Shift Workers: A Multi-Center Study

**DOI:** 10.3389/fpubh.2021.761279

**Published:** 2021-11-17

**Authors:** Juho Sim, Byung-Yoon Yun, Jiho Lee, Sung Kyung Kim, Seunghyun Lee, Ara Cho, Seunghan Kim, Chang-young Kim, Yeon Suh Oh, Jin-Ha Yoon

**Affiliations:** ^1^Department of Public Health, Graduate School, Yonsei University, Seoul, South Korea; ^2^Department of Preventive Medicine, Yonsei University College of Medicine, Seoul, South Korea; ^3^Department of Occupational and Environmental Medicine, University of Ulsan College of Medicine, Ulsan, South Korea; ^4^Department of Occupational and Environmental Medicine, Wonju Severance Christian Hospital, Wonju College of Medicine, Yonsei University, Wonju, South Korea; ^5^Institute of Occupational and Environmental Medicine, Yonsei University Wonju College of Medicine, Wonju, South Korea; ^6^Research Affairs of Yonsei University, Seoul, South Korea; ^7^Department of Occupational Health, Graduate School of Public Health, Yonsei University, Seoul, South Korea; ^8^BigData Center, Ulsan University Hospital, Ulsan, South Korea; ^9^Environmental Health Center, Ulsan University Hospital, University of Ulsan College of Medicine, Ulsan, South Korea; ^10^The Institute for Occupational Health, Yonsei University College of Medicine, Seoul, South Korea

**Keywords:** insomnia, consecutive night shifts, shift workers, common data model (CDM), medical examination data

## Abstract

**Objectives:** There is a need to determine the optimal limit of consecutive night shift work to reduce insomnia caused by the accumulation of sleep problems among night shift workers. This study aimed to investigate the prevalence of insomnia caused by consecutive night shifts and evaluate the night shift duration that worsens insomnia the most, using a large amount of medical examination data.

**Methods:** Night shift profiles and baseline demographics data of three hospitals were collected from January 2015 to December 2017. For subjects who had been examined more than once at the same institution, information corresponding to the most recent date was used. Multivariate logistic regression was performed to estimate odds ratios (ORs) and 95% confidence intervals (CIs). Pooled ORs were calculated by using the results of the three institutions.

**Results:** Of the 33,669 participants, 31.3% were female. The average age was 41.1 ± 11.1 years and the prevalence of insomnia was 38.7% (*n* = 13,025). After adjusting for potential confounders and compared to workers who reported not working in consecutive night shifts, odds of insomnia were greatest among workers reporting working three consecutive nights (OR 2.65, 95% CI 1.97–3.56) followed by those working two nights (OR 1.81, 95% CI 1.45–2.26), five nights (OR 1.78, 95% CI 1.56–2.03), and four nights (OR 1.68, 95% CI 1.55–1.82).

**Conclusion:** Our study demonstrates a significant relationship between consecutive night shift and insomnia with multicenter examination data, using common data model. This study could be a basis for establishing policies and guidelines that improve night shift workers' health.

## Introduction

Shift work is a working system used by 15–20% of employers worldwide as a way to provide continuous production or service every day (1). The change in working hours due to shift work was a social change related to the expansion in service industries (2). According to a survey conducted in 2015, 19% of workers worked at night in Europe (3), and approximately 40% of workers in the healthcare field of the European Union had shift work (4). In Republic of Korea, ~15.5% of all 89,582 companies worked shifts including night shifts (5).

Many studies have shown that shift work, including night shifts, was associated with the occurrence of various diseases. Shift work showed a significant correlation with cardiovascular disease, breast cancer, digestive disorders, arthritis, attention deficit, and fatigue as a consequence of mental and sleep problems (6–13). Working night shifts is associated with short sleep time, and causes sleep disorders such as insomnia and drowsiness (14, 15). Furthermore, insomnia, which is related to night shifts, has been shown to cause hypertension (16). According to a study of electronic manufacturers' employees in Republic of Korea, shift work was associated with insomnia, depression, and suicidal thoughts, respectively (17).

Various factors could be considered when studying the relationship between night shift work and sleep disorders, including insomnia. One study implied that nurses having <11 h between shifts showed significant positive association with insomnia, excessive sleepiness, and excessive fatigue (18). Another study revealed that fixed night shifts were more related to sleep and mental health problems than fixed day, rotating day, and rotating night shifts (19). Studies like these, which should be continuously performed, could help improve the health of night shift workers by providing basic data to be used as a management guideline for those workers.

Consecutive night shift can be a risk factor for various health problems. A quasi-experimental crossover study of police officers, showed that continuous night work shortened sleep time and caused deterioration of sleep quality, and there was an increase in sleep debt with longer consecutive night shifts (20). Therefore, there is a need for a study on the optimal limit of consecutive night shift work to reduce the fatigue caused by the accumulation of sleep problems among night shift workers.

Thus, this study aimed to investigate the prevalence of insomnia according to consecutive night shifts and to provide basic data for finding the safest consecutive night shift duration, using a large medical examination data.

## Methods

### Study Population

Korean Workers Health Examination-Common Data Model (KWHE-CDM) was applied for five medical institutions that conduct special health checkups, two of which participated in this study. Data of the KWHE-CDM consists of general measurement, common questionnaires, special questionnaires, and night shift questionnaires. Data on night shift and baseline demographics were collected from January 2015 to December 2017. For subjects who had been examined more than once at the same institution, information corresponding to the most recent date was used.

In total, 13,311 workers from Sinchon Severance Hospital, 6,429 workers from Wonju Severance Hospital, and 13,929 workers from Ulsan University Hospital (33,669 workers) were recruited. The same analysis was conducted at each medical institution with the same statistic syntax so that equivalent results were calculated using the CDM method.

### Procedure

The primary outcome of this study was the presence of insomnia. Insomnia was measured using the Insomnia Severity Index (ISI) questionnaire, which has been broadly used as a reliable scale of insomnia. ISI is a survey tool used to quantify insomnia severity (21) and has been proven to be valid in Republic of Korea (22). Each question of the ISI is rated by a 5-point Likert scale, yielding a total score ranging from 0 to 28. Based on the score, the participants were categorized into four groups as follows: 0–7 (absence of insomnia), 8–14 (sub-threshold insomnia), 15–21 (moderate insomnia), and 22–28 (severe insomnia) (23). Those with scores ranging from 0–7 were classified into the non-insomnia group, and those in the other score categories (sub-threshold, moderate, and severe insomnia) were classified into the insomnia group.

Data on night shift work were collected from the participants, using the night shift questionnaires in the CDM. The number of consecutive night shifts was collected through a question “How many consecutive night shifts did you usually work in the past year?” using a 5-point Likert scale with the following answers: none, 2 nights, 3 nights, 4 nights, and ≥5 nights, respectively.

Covariates used in the multivariate analysis were obtained from baseline demographics (age and sex) and night shift profiles including shift type, shift interval, and working hours. Shift type at current workplace was assessed, and the answers were categorized as follows: 3 shifts, 2 shifts, every other day, fixed, and irregular. Workers whose rest time between shifts was <11 h were classified into the “quick return” group; others were classified into “slow return” group. Whether workers worked 52 or more hours per week on average or not was used as a working-hour covariate.

### Statistical Analysis

For continuous and categorical data, differences between participants with and without insomnia were evaluated using the independent *t*-test and the chi-square test, respectively. Odds ratios (ORs) of insomnia with 95% confidence intervals (CIs) were estimated using a multiple logistic regression model. The association between shift work and insomnia was explored using multivariable logistic regression, and OR and 95%CI were reported. Model 1 was adjusted for sex and age, Model 2 was adjusted for sex, age, working hours, and rest time between shifts. The data of each hospital were analyzed with equivalent statistical methods. Based on the logistic regression results of each organization, meta-analysis was implemented to confirm the integrated results, and a random effect model was applied to “consecutive night shift” to further implement subgroup meta-analysis. The weight acquired through the standard error was used to generate pooled ORs and 95% CIs for insomnia.

All statistical tests were two-sided, and statistical significance was defined as a *p* < 0.05. The R software version 4.0.3 (R Foundation for Statistical Computing, Vienna, Austria) was used for all statistical analyses.

### Ethics Statement

The study protocol was approved by Severance Hospital's Institutional Review Board and followed the ethical requirements of the 1975 Declaration of Helsinki (IRB: Y-2020-0011). Because of the retrospective nature of this study, informed permission from the participants was waived.

## Results

The baseline characteristics of the entire participants are summarized in Table 1. In total, 33,669 participants were recruited in this study, with an average age of 41.1 ± 11.1 years. The proportion of females was 31.3%, and the prevalence of insomnia was 38.7% (*n* = 13,025).

**Table 1:** Baseline characteristics of the entire participants stratified by insomnia.

	**Total**	**Insomnia**	**Non-insomnia**	***P*-value**
	**(*N* = 33,669)**	**(*N* = 13,025)**	**(*N* = 20,644)**	
**Sex**				
Male	23,140 (68.7%)	8,391 (64.4%)	14,749 (71.4%)	<0.001
Female	10,529 (31.3%)	4,634 (35.6%)	5,895 (28.6%)	
**Age**				
Mean ± SD	41.1 ± 11.1	40.4 ± 10.8	42.2 ± 11.2	<0.001
**Working hours**				
Under 52 h	24,595 (73.0%)	9,389 (72.1%)	15,206 (73.7%)	0.002
Over 52 h	9,074 (27.0%)	3,636 (27.9%)	5,438 (26.3%)	
**Rest time between shifts**				
Slow return (11 h or more)	26,094 (77.5%)	9,817 (75.4%)	16,277 (78.8%)	<0.001
Quick return (<11 h)	7,575 (22.5%)	3,208 (24.6%)	4,367 (21.2%)	
**Consecutive night shifts**				
None	7,238 (21.5%)	1,955 (15.0%)	5,283 (25.6%)	<0.001
2 nights	3,274 (9.7%)	1,331 (10.2%)	1,943 (9.4%)	
3 nights	4,041 (12.0%)	2,095 (16.1%)	1,946 (9.4%)	
4 nights	5,066 (15.0%)	1,983 (15.2%)	3,083 (14.9%)	
5 or more nights	14,050 (41.7%)	5,661 (43.5%)	8,389 (40.6%)	

The workers with and without insomnia were 13,025 and 20,644, respectively. The proportion of women in insomnia group was 35.6% and that in the non-insomnia group was 28.6%, statistically significant differences (*p* < 0.001). The average age in insomnia group was 40.4 ± 10.8 years, 1.8 years younger than 42.2 ± 11.2 years in non-insomnia group, with a statistically significant difference (*p* < 0.001). Compared to workers with non-insomnia, those with insomnia were more likely to be female, having rest time between shifts of <11 h (“quick return”), and having five or more consecutive night shifts (*p* < 0.01 for all).

Pooled ORs were calculated by using the results of the three institutions in Table 2. The factors affecting insomnia were sex, age, quick return, and consecutive night shift in all institutions. Model 1 was adjusted using age and sex as covariates. The final adjusted model used age, sex, working hour and quick return as covariates. In Model 2, all the answers regarding consecutive night shifts were significantly associated with insomnia compared with the “none.” Additionally, “3 nights” had the highest OR of 2.65 (95% CI 1.97–3.56), followed by 2 nights, 5 nights, and 4 nights [2 nights: OR 1.81 (95% CI 1.45–2.26); 5 or more nights: OR 1.78 (95% CI 1.56–2.03); 4 nights: OR 1.68 (95% CI 1.55–1.82)].

**Table 2:** Pooled odds ratios of insomnia in multivariable logistic regression models.

	**Model 1**	**Model 2**
**Consecutive night shifts**		
None	(Reference)	(Reference)
2 nights	1.69 (1.29–2.21)	1.81 (1.45–2.26)
3 nights	2.32 (1.59–3.39)	2.65 (1.97–3.56)
4 nights	1.78 (1.64–1.93)	1.68 (1.55–1.82)
5 or more nights	1.78 (1.54–2.06)	1.78 (1.56–2.03)

*Adjusted for Model 1: sex, age*.

*Adjusted for Model 2: sex, age, working hours, Rest time between shifts*.

**Figure 1:**
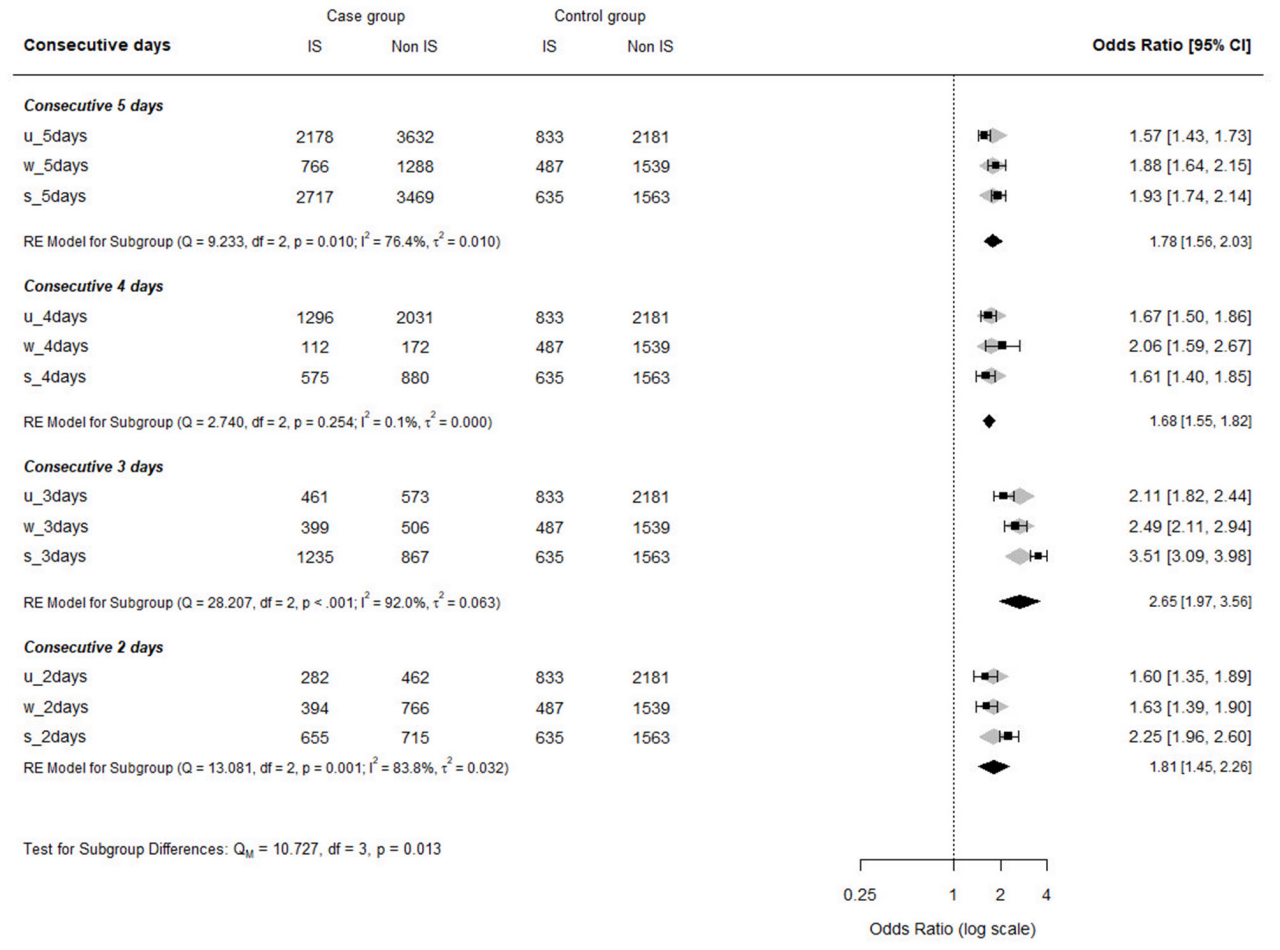
Odds ratios for prevalence of insomnia by consecutive night shift in each institution. IS, insomnia; u, Ulsan University; s, Severance; w, Wonju Severance.

The ORs of insomnia by consecutive night shifts in each institution are shown in Figure 1. Regardless of the number of consecutive night work days, all institutions showed higher OR values compared to those who did not. Moreover, all three institutions showed the highest OR in workers with 3 nights. The completed version of the baseline characteristics and the multivariable logistic regression model of the institutions is summarized in Supplementary Table 1.

## Discussion

According to the results of this study, consecutive night shift was significantly associated with the presence of insomnia. This relationship was significant even after adjusting for covariates including age, sex, working hours, and quick return. Although a few studies have elucidated the relationship between consecutive night shift and insomnia, many people still worked night shifts for five consecutive nights or more on average in this study.

In a study of Korean nurses, the incidence of insomnia increased as the number of consecutive night shifts increased (24); however, in this study, the OR for insomnia was highest at “3 nights” compared to “none.” When consecutive night shift was implemented, although the quality of sleep and the duration of sleep were shortened, the difference was not significant on days other than the last day, even with an increase in the number of consecutive night shifts and the presence of accumulated lack of sleep on the last day (25). Although consecutive night shift is an important factor in insomnia, insomnia does not seem to increase as the number of consecutive night shifts increases. Since it is well-known that insomnia is associated with the development of various diseases including hypertension, diabetes, cardiovascular diseases and mortality, consecutive night shifts should be adjusted, considering the health effects on workers (6, 7, 16).

In our study, working three consecutive night shifts had the highest OR for insomnia compared to others. Bjorn et al. conducted a study of adaptation and readaptation of night work on oil Rig night workers. The study implies that more consecutive night shifts induce circadian rhythm adaptation, resulting in better and longer sleep throughout the day (26). On the other hand, according to a study of police officers, sleep length adaptation did not occur even after six consecutive night shifts, and last day fatigue is the most difficult to recover from regardless of the number of consecutive shifts (20). This implies that workers with 3 consecutive shifts were more repetitively exposed that situation, which could result in higher OR of insomnia. Considering that short-term consecutive night shifts result in frequent fatigue and the effect of long-term continuous night shift on sleep is unclear, it is necessary to establish accurate standards for consecutive night shifts, considering sleep health. Another explanation is linked with a healthy worker effect. Healthy worker effect indicates that healthier workers could selectively survive in harsh working environment (27). In our study, it is plausible that healthy workers who have already adapted to the harsh environment, which indicates 4 or 5 consecutive night shifts, might remain in the workplace, thus OR of insomnia could be underestimated.

Danish police officers preferred four consecutive night shifts to seven or two (28). The preferred number of consecutive night shifts in Republic of Korea has not been investigated, but it is expected that this number should depend on an individual's ability to recover. Shift work schedules varies according to economic incentives and family/individual life (29), and it was discovered that shift work schedules were established with family life as the primary consideration (30). Therefore, when adjusting the schedule for health, it will be impossible to do only the schedule that the individual wants. Further studies should be implemented to elucidate the appropriate number of consecutive night shifts with respect to workers' health.

This study has several strengths. First, using the CDM method for multiple institutions, many multicenter participants were enrolled. The same night shift questionnaires were used at each hospital and the data were standardized into an identical structure. Second, analysis was performed with verified variables, and validated survey of insomnia was used as an outcome. Through ISI, a valid assessment of insomnia could be made. Working year, working hours, short return, and consecutive night shift were all validated with several studies, as most workers in Republic of Korea answered the same questions (31, 32). Third, because we enrolled participants from various occupational field, the health effects of consecutive night shift on shift workers makes this study generalizable to various workers in other fields.

There are also some limitations in this study. First, lifestyle factors including drinking history, smoking history were not adjusted, which could affect the quality of sleep. Second, history of sleep disorders or related diseases including thyroid disease, psychiatric disease were not clarified in this study due to lack of data. It might be better to exclude or adjust for diseases related to insomnia if possible. Third, the exact profile of night shift was not provided. The number of consecutive shifts or shift intervals were not provided and the average trend in the questionnaire were used. Finally, this is a cross-sectional study and a causal relationship could not be established. However, insomnia symptoms were reported in the health examination data based on the symptoms in the last 2 weeks, whereas the questionnaires on night shift intensity reported data obtained 6-month prior to the survey date. Since exposure occurred before health consequence, some degree of temporal relationship could be achieved from this study. Therefore, causal relationship between consecutive night shifts and insomnia should be surely further studied in the future.

In conclusion, our study elucidates a significant relationship between consecutive night shift and insomnia using the CDM method with multicenter examination data. This study could be a basis for establishing policies and guidelines that improve night shift workers' health. Further studies should be performed to identify the causal relationship of consecutive night work and insomnia based on this study.

## Data Availability Statement

The original contributions presented in the study are included in the article/Supplementary Material, further inquiries can be directed to the corresponding author/s.

## Ethics Statement

The studies involving human participants were reviewed and approved by Severance Hospital's Institutional Review Board. Written informed consent for participation was not required for this study in accordance with the national legislation and the institutional requirements.

## Author Contributions

JS has written the manuscript and responsible for the conception and data analyses in cooperation with B-YY, JL, C-yK, YO, SKK, and J-HY has collected data each institution. SL, AC, and SK have contributed with literature review. J-HY has contributed with insight, scientific discussion, and editing of the manuscript. All authors contributed to the article and approved the submitted version.

## Funding

This work was supported by the Republic of Korea Health Industry Development Institute through Social and Environmental Risk Research funded by the Ministry of Health & Welfare (HI19C0052).

## Conflict of Interest

The authors declare that the research was conducted in the absence of any commercial or financial relationships that could be construed as a potential conflict of interest.

## Publisher's Note

All claims expressed in this article are solely those of the authors and do not necessarily represent those of their affiliated organizations, or those of the publisher, the editors and the reviewers. Any product that may be evaluated in this article, or claim that may be made by its manufacturer, is not guaranteed or endorsed by the publisher.
