# Supplementary material for: The Association Between the Number of Consecutive Night Shifts and Insomnia Among Shift Workers: A Multi-Center Study
Source: Front Public Health. 2021 Nov 17;9:761279. doi: 10.3389/fpubh.2021.761279 (PMC8637843; doi:10.3389/fpubh.2021.761279)
Supplement: Supplementary file 1 [file Table_1.DOCX]

Supplementary Material

**Supplementary Table 1** Baseline characteristics and Multivariable Logistic Regression Models of Each Institution

| **Institution #1** | **Insomnia**  **(N=5817)** | **Non Insomnia**  **(N=7494)** | **P-value** | **Model 1** | **Model 2** |
| --- | --- | --- | --- | --- | --- |
| (Intercept) |  |  |  | 0.531 (0.448-0.629) | 0.463 (0.383-0.561) |
| Sex |  |  |  |  |  |
| Male | 2707 (46.5%) | 4022 (53.7%) | <0.001 | (reference) | (reference) |
| Female | 3110 (53.5%) | 3472 (46.3%) |  | **1.089 (1.007-1.179)** | **1.120 (1.033-1.214)** |
| Age |  |  |  | **0.991 (0.988-0.995)** | **0.992 (0.989-0.996)** |
| Mean±SD | 36.8±10.0 | 38.1±10.2 | <0.001 |  |  |
| Median [Min, Max] | 34.0 [19.0, 69.0] | 36.0 [19.0, 69.0] |  |  |  |
| Working hours |  |  |  |  |  |
| under 52h | 4088 (70.3%) | 5224 (69.7%) | 0.490 |  | (reference) |
| over 52h | 1729 (29.7%) | 2270 (30.3%) |  |  | 0.998 (0.915-1.088) |
| Rest time between shifts |  |  |  |  |  |
| Slow return (more 11hr) | 4259 (73.2%) | 5767 (77.0%) | <0.001 |  | (reference) |
| Quick return (< 11hr) | 1558 (26.8%) | 1727 (23.0%) |  |  | **1.295 (1.188-1.412)** |
| Consecutive night shifts |  |  |  |  |  |
| None | 635 (10.9%) | 1563 (20.9%) | <0.001 | (reference) | (reference) |
| 2 nights | 655 (11.3%) | 715 (9.5%) |  | **2.176 (1.187-2.508)** | **2.182 (1.891-2.516)** |
| 3 nights | 1235 (21.2%) | 867 (11.6%) |  | **3.301 (2.898-3.760)** | **3.392 (2.973-3.871)** |
| 4 nights | 575 (9.9%) | 880 (11.7%) |  | **1.734 (1.502-2.001)** | **1.852 (1.596-2.148)** |
| 5 or more nights | 2717 (46.7%) | 3469 (46.3%) |  | **2.006 (1.804-2.231)** | **2.056 (1.847-2.289)** |

| **Institution #2** | **Insomnia**  **(N=2158)** | **Non Insomnia**  **(N=4271)** | **P-value** | **Model 1** | **Model 2** |
| --- | --- | --- | --- | --- | --- |
| (Intercept) |  |  |  | 0.716 (0.570-0.898) | 0.566 (0.445-0.720) |
| Sex |  |  |  |  |  |
| Male | 1311 (60.8%) | 2782 (65.1%) | <0.001 | (reference) | (reference) |
| Female | 847 (39.2%) | 1489 (34.9%) |  | 1.034 (0.921-1.160) | 1.115 (0.990-1.257) |
| Age |  |  |  | **0.982 (0.978-0.987)** | **0.983 (0.979-0.988)** |
| Mean±SD | 41.4±12.0 | 44.8±12.5 | <0.001 |  |  |
| Median [Min, Max] | 40.0 [20.0, 77.0] | 45.0 [20.0, 85.0] |  |  |  |
| Working hours |  |  |  |  |  |
| Under 52hrs | 1182 (54.8%) | 2588 (60.6%) | <0.001 |  | (reference) |
| Over 52hrs | 976 (45.2%) | 1683 (39.4%) |  |  | **1.368 (1.221-1.533)** |
| Rest time between shifts |  |  |  |  |  |
| Slow return (more 11hr) | 1563 (72.4%) | 3338 (78.2%) | <0.001 |  | (reference) |
| Quick return (< 11hr) | 595 (27.6%) | 933 (21.8%) |  |  | **1.181 (1.044-1.335)** |
| Consecutive night shifts |  |  |  |  |  |
| None | 487 (22.6%) | 1539 (36.0%) | <0.001 | (reference) | (reference) |
| 2 nights | 394 (18.3%) | 766 (17.9%) |  | **1.549 (1.315-1.826)** | **1.578 (1.336-1.862)** |
| 3 nights | 399 (18.5%) | 506 (11.8%) |  | **2.053 (1.711-2.464)** | **2.114 (1.759-2.539)** |
| 4 nights | 112 (5.2%) | 172 (4.0%) |  | **1.915 (1.475-2.486)** | **1.966 (1.511-2.557)** |
| 5 or more nights | 766 (35.5%) | 1288 (30.2%) |  | **1.761 (1.534-2.023)** | **1.668 (1.449-1.919)** |

| **Institution #3** | **Insomnia**  **(N=5050)** | **Non Insomnia**  **(N=8879)** | **P-value** | **Model 1** | **Model 2** |
| --- | --- | --- | --- | --- | --- |
| (Intercept) |  |  |  | 0.415 (0.350-0.492) | 0.395 (0.332-0.469) |
| Sex |  |  |  |  |  |
| Male | 4373 (86.6%) | 7945 (89.5%) | <0.001 | (reference) | (reference) |
| Female | 677 (13.4%) | 934 (10.5%) |  | **1.284 (1.118-1.475)** | **1.309 (1.139-1.504)** |
| Age |  |  |  | 0.997 (0.994-1.001) | 0.997 (0.994-1.000) |
| Mean±SD | 43.0±11.3 | 43.8±11.5 | <0.001 |  |  |
| Median [Min, Max] | 44.0 [19.0, 76.0] | 45.0 [18.0, 73.0] |  |  |  |
| Working hours |  |  |  |  |  |
| Under 52hrs | 4119 (81.6%) | 7394 (83.3%) | 0.0111 |  | (reference) |
| Over 52hrs | 931 (18.4%) | 1485 (16.7%) |  |  | **1.155 (1.051-1.269)** |
| Rest time between shifts |  |  |  |  |  |
| Slow return (more 11hr) | 3995 (79.1%) | 7172 (80.8%) | 0.0188 |  | (reference) |
| Quick return (< 11hr) | 1055 (20.9%) | 1707 (19.2%) |  |  | **1.151 (1.054-1.258)** |
| Consecutive night shifts |  |  |  |  |  |
| None | 833 (16.5%) | 2181 (24.6%) | <0.001 | (reference) | (reference) |
| 2 nights | 282 (5.6%) | 462 (5.2%) |  | **1.401 (1.170-1.676)** | **1.396 (1.166-1.670)** |
| 3 nights | 461 (9.1%) | 573 (6.5%) |  | **1.796 (1.524-2.115)** | **1.790 (1.519-2.109)** |
| 4 nights | 1296 (25.7%) | 2031 (22.9%) |  | **1.712 (1.538-1.905)** | **1.785 (1.601-1.990)** |
| 5 or more nights | 2178 (43.1%) | 3632 (40.9%) |  | **1.610 (1.461-1.773)** | **1.599 (1.451-1.762)** |
